# Examining the rural–urban differentials in yoga and mindfulness practices among middle-aged and older adults in India: secondary analysis of a national representative survey

**DOI:** 10.1038/s41598-023-49388-4

**Published:** 2023-12-13

**Authors:** Umakanta Sahoo, Santosh K. Sharma, Harshita Chari, Soumya Ranjan Nayak, Waad Ali, T. Muhammad

**Affiliations:** 1 Department of Statistics, Sambalpur University, Jyoti Vihar, Burla, Odisha India; 2 International Institute for Population Sciences, Mumbai, India; 3 Gokhale Institute of Politics and Economics, Pune, India; 4 Model Rural Health Research Unit, ICMR-RMRCBB, Tigiria, Odisha India; 5 Department of Geography, Sultan Qaboos University, Muscat, Oman; 6 Center for Healthy Aging, The Pennsylvania State University, University Park, USA

**Keywords:** Disease prevention, Geriatrics, Health services, Public health

## Abstract

Physical activity and mental well-being play an important role in reducing the risk of various diseases and in promoting independence among older adults. Appropriate physical activity, including yoga and mindfulness practices, can help rectify the loss of independence due to aging and have a positive influence on physical health and functional activities. This study assessed rural–urban differences in yoga and mindfulness practices and their associated factors among middle-aged and older Indian adults. The total sample size considered for the current analysis was 72,250 middle-aged and older adults (aged ≥ 45 years). Bivariate and multivariable logistic regression analyses were used to estimate the prevalence of yoga and mindfulness practices and examine the associations of selected variables with yoga and mindfulness practices among the participants. Further, we used the Fairley decomposition technique to determine the factors contributing to rural–urban differences in the prevalence of yoga and mindfulness practices among middle-aged and older adults. More than 9% of middle-aged and older adults in rural areas and 14% in urban areas reported practicing yoga and mindfulness activities more than once per week. Adults aged ≥ 65 years were more likely to practice yoga and mindfulness activities than those who age 45–54 years were. Those with an education of ten years and above were 2.3 and 2.1 times higher likely to practice yoga in rural (AOR: 2.28; CI: 2.07–2.52) and urban (AOR: 2.13; CI: 1.91–2.37) areas compared to their uneducated peers, respectively. The largest contributors in diminishing the gap in yoga practice among participants were education (44.2%), caste (2.5%), chronic diseases such as hypertension (4.53%), diabetes (1.71%), high cholesterol (3.08%), self-reported pain (5.76%), and difficulties in instrumental activities of daily living (1.22%). The findings suggest that middle-aged and older adults in urban areas practice yoga and mindfulness activities more than their peers in rural areas do. Education level, household characteristics, and health outcomes such as chronic conditions, pain, and functional difficulties explain the observed differences in yoga and mindfulness practices across rural and urban areas. Age-appropriate healthy practices such as yoga and mindfulness should be encouraged to enhance the physical and mental well-being of middle-aged and older adults, especially in rural areas.

## Introduction

Like several other emerging economies, India has undergone significant spatial transformation. These changes are reflected in the rise of the urban population, which has increased from 26% in 1990 to 36% in 2022, whereas the rural population has declined from 74% in 1990 to 64% in 2022^1^. Despite these shifts, the annual urban population growth rate in India has decreased from 3% in 1990 to 2% in 2022^1^. Another key demographic trend shaping India's demographic landscape is a growing aging population. With the increase in the aging population of India, there is also a challenge from a public health perspective in terms of the increased risk of chronic diseases and disability. According to the Population Census of India, 2011, there are nearly 104 million older adults (aged 60 years or above) in India, comprising 53 million females and 51 million males^2^. The size and share of older adults in the country has increased from 5.6% in 1961 to 8.6% in 2011^2^ and is projected to increase to 20% by 2050^3^.

It is well known that physical activity and mental well-being play an important role in reducing the risk of diseases and promoting independence in the older age^4,5^. Studies have report that physical inactivity stimulates overweight, impairs bodily function, and increases the incidence of noncommunicable diseases (NCDs)^4,6,7^. Age-related factors affect changes in the sensorimotor and neuromuscular systems, thus negatively affecting performance in static and dynamic postural control even in healthy older adults, leading to an increased risk of falls^8–11^. Thus, evidence suggests, appropriate physical activity throughout life is the key requirement of healthy ageing and a predictor of reduced mortality^12,13^. Therefore, it is imperative to address interventions for physical inactivity, especially among aging adults, in light of a recent study in India that revealed that 43.1% of individuals aged 60–69 years were physically inactive, and among those aged ≥ 70 years, the prevalence was even higher (63.3%)^14^. These findings emphasize the significance of developing interventions or preventative measures to mitigate physical inactivity, particularly among older adults who report a considerable share of NCDs. Despite understanding the various ailments that can trouble older adults, there is an increased prevalence of physical inactivity among older people, especially in low- and middle-income countries, including India^14,15^, and particularly in rural areas^16–18^, indicating the need for more effective strategies to promote physical activity in this population.

The term ‘yoga’ originates from ancient Indian philosophy having root in Sanskrit word ‘yuj’ meaning union, a union of the body, mind, and soul. It is also a systematic psychological practice of improving awareness and realizing about oneself. Yoga comprises different components, including asana (postures), pranayama (breathing), and dhyana (meditation or yogic mindfulness), that can cultivate physical activity, relaxation, and self-awareness. Yoga can provide therapeutic benefits for both physical and mental well-being. It enhances physical flexibility, coordination, and strength, whereas breathing practices and meditation help calm down and focus on developing greater awareness and diminishing anxiety. Yoga plays an important role in reducing the risk of lifestyle diseases and promoting independence in older age as it affects both physical and mental health. Practicing yoga improves overall physical function, reduces stress or anxiety, enriches sleep quality, promotes healthy eating habits, and enhances calmness^19^. The practice of some yoga postures can help prevent, stabilize, or reduce the severity of cardiovascular diseases, including blood pressure and coronary heart disease, improve pulmonary performance, help control weight loss, and reduce glucose tolerance^20,21^. A prospective cohort study reported that adjunct naturopathy and yoga can control body glucose levels and reduce the overall need for antidiabetic medications^22^.

As most older adults, particularly those in rural areas, can have inactive lifestyles owing to their low participation in social activities and the unavailability of age-appropriate recreational facilities^17,23^, or limited participation in light- and low-intensity household activities^24,25^, yoga can be an appropriate and comprehensive intervention to promote active and healthy lifestyles. With yoga being available through mass communication networks in recent times, there has been an observable increase among older adults who have started practicing yoga^26,27^. However, there is a lack of information on rural and urban differences in yoga and mindfulness practices across the country, which would help us to recognize areas where there is a need for intervention. Hence, this study assessed differences in the practice of mindfulness activities, such as yoga, meditation, and pranayama, among middle-aged and older adults in India. It also assessed the major factors that can contribute to reducing rural–urban differences in these practices.

### Working hypotheses

There are significant urban–rural differences in the practice of yoga/meditation/pranayama and other mindfulness activities among middle-aged and older adults in India.

Socio-demographic (age, sex, education, marital status, living arrangement, caste, religion, and household wealth) and health-related factors (NCDs, pain, functional ability, and self-perceived health) are associated with yoga and mindfulness practices differently in rural and urban areas.

## Methods

### Data

**Figure 1:**
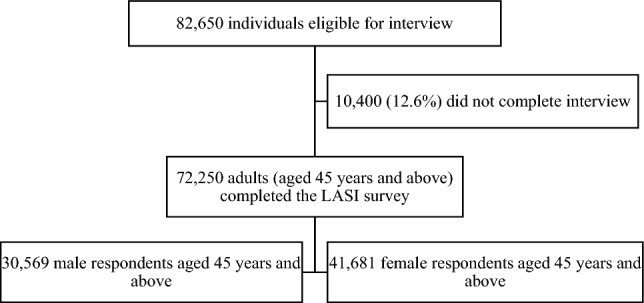
Sample selection criteria for this study.

This study used unit-level data from a large-scale population-based survey of the Longitudinal Aging Study in India (LASI) Wave 1, conducted between 2017–18. LASI is a nationally representative study of the health, economic, and social well-being of adults aged 45 years and above (and their spouses, irrespective of age). This collaborative study involved organizations such as the International Institute for Population Sciences (IIPS), Mumbai, and Harvard T.H. Chan School of Public Health and the University of Southern California, USA. Details of the sampling procedure, instruments used, and findings of the survey are available in the national report^28^. Age was the only selection criterion in this study, and 72,250 middle-aged and older adults aged ≥ 45 years were selected for the study (Fig. 1).

### Variables of the study

### Outcome variable

Yoga and mindfulness practices were assessed using the question “How often do you engage in any of the following activities like yoga, meditation, asana, pranayama or similar?” The variable was recoded as yes if the respondent reported practicing yoga, meditation, asana, pranayama, etc., more than once a week, and otherwise no.

### Independent variables

We used a set of independent variables in the analysis. These independent variables were age groups in years (45–54, 55–64, 65 +), sex of the respondent (male, female), education (no education, less than 5 years, 5–9 years completed, 10 or more years completed), marital status (currently married, widowed, others), living arrangements (living alone, living with spouse, living with spouse and children, living with children and/or others), religion (Hindu, Muslim, Christian, Others), caste group (Scheduled Caste [SC], Scheduled Tribe [ST], Other Backward Caste [OBC], Other), Monthly Per capita Consumption Expenditure (MPCE) quintile (poorest, poorer, middle, richer, richest), currently working status (currently working, ever worked but currently not, never worked), self-reported NCDs: Hypertension or high blood pressure, Diabetes or high blood sugar, Cancer or a malignant tumour, Chronic lung disease such as asthma, Chronic Obstructive Pulmonary Disease (COPD)/bronchitis, Chronic heart diseases/congestive heart failure/other chronic heart problems, stroke, Arthritis or rheumatism, Osteoporosis or other bone/joint diseases, Any neurological/psychiatric problems such as depression, Alzheimer’s/Dementia, unipolar/bipolar disorders, convulsions, Parkinson’s etc., and High cholesterol, self-reported pain, self-rated health (good and poor)^29^, and difficulty in activities of daily living (ADL) and instrumental ADL (reporting difficulty in at least one activity).

### Statistical methods

Descriptive statistics and bivariate analysis were used to understand the factors associated with yoga practice among middle-aged and older Indian adults. Binary logistic regression was used to determine the factors associated with practicing yoga/meditation/asana/pranayama among middle-aged and older adults. The basic form of the logistic regression model, which yields the probability of the occurrence of an event, can be expressed as

$${\log_{\rm e} }[{\text{P}} ({\text{Y}}_{\rm i}=1|{\text{X}}_{\rm i}) / 1-{\text{P}}({\text{Y}}_{\rm i}=1|{\text{X}}_{\rm i})]={{{\log}}}_{{{\rm e}}}\left[\uppi |1-\uppi \right]={\upalpha }+\upbeta_{1}{\text{X}}_{\rm i1},\ldots {\upbeta_{\rm k}} {\text{X}}_{\rm ik}$$

where Y_i_ is the binary response variable and X_i_ is the set of explanatory variables such as socio-demographic characteristics, and β_1_, β_2_… β_k_ are the coefficients of the X_i_ variables.

We used an extension of the Blinder-Oaxaca technique, which is appropriate for binary models, to decompose the rural–urban gap in the prevalence of practicing some form of yoga among middle-aged and older adults more than once a week into contributions that can be attributed to different factors. According to Fairlie, this technique was the first relatively simple method to use logit and probit estimates to describe the black/white gap in self-employment in 1999^30^. These nonlinear procedures are similar to Blinder and Oaxaca linear decomposition^31^. For similarity, both methods decompose the respective differences or gaps into observable and unobservable features. However, both procedures have been used in different frameworks. Although the Blinder-Oaxaca technique is used in linear models, the Fairlie technique is suitable for nonlinear models. All statistical analyses were conducted using Stata version 16^32^ using weighted individual variables in the dataset.

## Results

### Sample profile

Table 1 shows the sample profiles of middle-aged and older adults in India, based on the selected variables. There was a higher percentage (42.6%) of adults age group 45–54 years, followed by older adults aged 65 + years (29.5%). Approximately 57.7% of participants were females. Around 46% of the older adults had no formal education and 19.5% had more than 10 years of education. Of the older adults, 76.7% were currently in a marital union and 20.2% were widowed. There were about 60.4% older adults who lived with their spouses and children, whereas 3.2% older adults lived alone. Among the study participants, there was a high percentage of Hindus (73.3%), followed by Muslims (12.0%), Christians (10.0%), and others (4.7%).

**Table 1:** Sample profile of study variables used for older adults in the analysis, LASI, 2017–18.

Background characteristics	N	%
Age (in years)		
45–54	30,782	42.6
55–64	20,136	27.9
65+	21,332	29.5
Sex		
Male	30,569	42.3
Female	41,681	57.7
Education		
No education	33,212	46.0
Less than 5 years complete	80,054	11.1
5–9 years complete	16,910	23.4
10 or more years complete	14,074	19.5
Marital status		
Currently Married	55,396	76.7
Widowed	14,593	20.2
Others	2257	3.1
Living arrangement		
Living alone	2313	3.2
Living with spouse	10,838	15.0
Living with spouse and children	43,663	60.4
Living with children and /or others	15,436	21.4
Religion		
Hindu	52,973	73.3
Muslim	8667	12.0
Christian	7215	10.0
Others	3390	4.7
Caste		
SC	12,046	16.7
ST	12,509	17.3
OBC	27,184	37.7
Others	20,398	28.3
Current work status		
Currently Working	32,990	45.7
Ever worked but currently not	17,951	24.9
Never Worked	21,289	29.5
MPCE quintile		
Poorest	14,158	19.6
Poorer	14,530	20.1
Middle	14,537	20.1
Richer	14,686	20.3
Richest	14,339	19.8
Total	72,250	100.0

SC: Scheduled caste; ST: Scheduled tribe; MPCE: Monthly Per capita Consumption Expenditure.

Table 2 presents the prevalence estimates of self-reported morbidities among the older adults in India. Among self-reported chronic conditions, the most prevalent were hypertension or high blood pressure (26.3%), arthritis or rheumatism, osteoporosis or other bone/joint diseases (15.6%), and diabetes or high blood sugar (11.5%). Approximately 35.5% of the elderly reported pain and 19% reported poor health. When asked about difficulties in daily living activities, 16% reported difficulty in ADL and 36% reported difficulty in IADL.

**Table 2:** Sample profile of older adults in India according to self-reported morbidities, LASI 2017–18.

Background characteristics	% (N = 72,250)	95% CI
Self-reported chronic conditions		
Hypertension or high blood pressure	26.3	(25.5, 27.1)
Diabetes or high blood sugar	11.5	(10.8, 12.3 )
Cancer or a malignant tumour	0.6	(0.5, 0.7)
Chronic lung disease such as asthma, COPD, bronchitis	6.3	(5.8, 6.9)
Chronic heart diseases/congestive heart failure/other chronic heart problems	3.6	(3.2, 4.0)
Stroke	0.0	1.6, 2.0
Arthritis or rheumatism, Osteoporosis or other bone/joint diseases	15.6	(14.9, 16.4)
Any neurological/psychiatric problems such as depression, Alzheimer’s/Dementia, unipolar/bipolar disorders, convulsions, Parkinson’s etc.	2.4	(2.2, 2.6)
High cholesterol	2.2	(2.0, 2.4)
Self-reported Pain	35.5	(34.8, 36.2)
Self-rated health		
Good health	81.1	(80.4, 81.7)
Poor health	18.9	(18.3, 19.6)
Difficulty in daily living activities		
ADL	15.8	(15.2, 16.4)
IADL	35.9	(35.1, 36.7)

COPD: Chronic Obstructive Pulmonary Disease; ADL: Activities of daily living; IADL: Instrumental activities of daily living.

**Figure 2:**
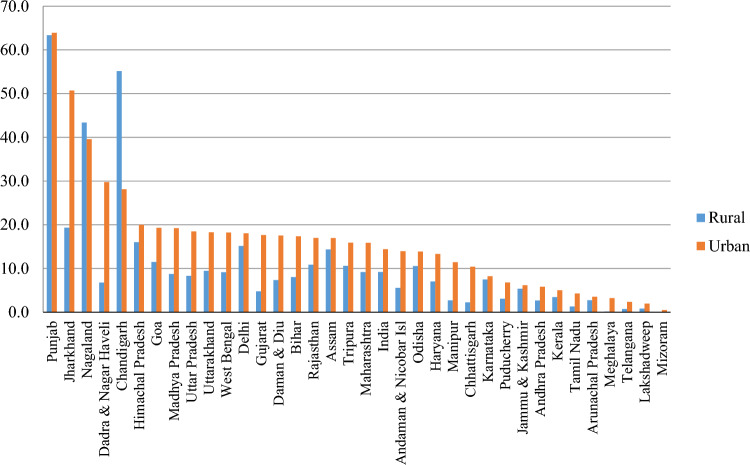
Rural–urban gap in practicing yoga or mediation or asana or pranayama more than once a week across the states among older adults in India, 2017–18.

Figure 2 presents the prevalence of yoga and mindfulness practices across different states and union territories of India, stratified by rural and urban areas. More than 9% of middle-aged and older adults in rural areas and 14% in urban areas reported practicing yoga and mindfulness activities more than once per week.

Table 3 presents the rural–urban differentials in the proportion of older adults practising yoga more than once a week in India. Approximately 9.2% of older adults residing in rural areas of India practiced yoga, compared to 14.4% in urban areas. A higher percentage of adults aged ≥ 65 years practiced yoga (9.3%) in rural areas. In urban areas, this percentage was higher among adults aged 55–64 years. Urban male older adults (16.2%) practicing yoga were more common than were rural older adults (10.6%). In both rural and urban areas, yoga practice increased with increasing levels of education, and was higher among adults with 10 or more years of education in both rural (19.9%) and urban areas (21.8%). A higher percentage of married older adults practiced yoga in both rural (9.9%) and urban areas (15.8%). Of those living with their spouses and children who practiced yoga, 10.2% resided in rural areas and 16% in urban areas. In both religion and caste, a higher percentage of other categories from rural areas (35.9%) and urban areas (25.3%) practiced yoga than Hindus, Muslims, and Christians did. In rural areas, a higher percentage of older adults who had never worked (11.5%) practiced yoga, whereas in urban areas, middle-aged and older adults who had worked, but were currently not working, had a higher proportion (16.5%) who practiced yoga. In rural areas, an increasing pattern of practicing yoga was observed across the MPCE quintile, whereas in urban areas it was higher in the middle strata of the MPCE quintile. Approximately 11.4% of older adults with at least one NCD practiced yoga in rural areas and 15.3% practiced yoga in urban areas.

**Table 3:** Rural–urban differential in the percentage of older adults practicing yoga/meditation/asana/pranayama more than once a week by background characteristics, India, 2017–18.

Background characteristics	Rural		Urban	
	%	N	%	N
Age (in years)				
45–54	9.14	19,141	13.37	11,340
55–64	9.19	13,115	16.60	6846
65+	9.26	13,928	14.06	7216
Sex				
Male	10.63	19,777	16.24	10,461
Female	8.12	26,407	13.21	14,941
Education				
No education	6.29	25,886	7.56	7065
Less than 5 years complete	9.85	5,407	10.05	2578
5–9 years complete	12.15	9,611	13.21	7141
10 or more years complete	19.86	5,280	21.84	8618
Marital status				
Currently married	9.85	35,641	15.79	19,236
Widowed	7.14	9,396	10.64	5076
Others	6.72	1,147	9.11	1088
Living arrangement				
Living alone	6.14	1,604	7.56	690
Living with spouse	8.51	7,160	15.05	3206
Living with spouse and children	10.20	27,968	16.03	15,659
Living with children and /or others	7.48	9452	10.67	5847
Religion				
Hindu	8.02	34,314	15.10	18,156
Muslim	11.00	4507	9.79	4074
Christian	4.16	5067	8.32	2109
Others	35.93	2294	25.29	1062
Caste				
SC	9.10	8603	10.30	3344
ST	5.02	9615	11.90	2806
OBC	8.54	17,317	12.08	9614
Others	12.57	10,587	19.37	9590
Current work status				
Currently working	8.16	23,254	12.72	9426
Ever worked but currently not	9.18	11,346	16.56	6448
Never worked	11.46	11,580	14.57	9524
MPCE quintile				
Poorest	5.62	9008	8.43	5015
Poorer	7.36	9237	13.56	5173
Middle	9.10	9283	18.13	5123
Richer	11.22	9415	16.01	5149
Richest	13.59	9241	16.50	4942
Total	9.19	46,184	14.41	25,402

SC: Scheduled caste; ST: Scheduled tribe; MPCE: Monthly Per capita Consumption Expenditure.

Table 4 presents the rural–urban differentials in the proportion of older adults who practiced yoga/meditation/asanas and pranayama more than once a week, according to their self-reported morbidities. Around 13% of adults from rural areas and 16% from urban areas who were diagnosed with hypertension or high blood pressure practiced yoga. A higher percentage of older adults from urban areas with arthritis or rheumatism, osteoporosis, or other bone/joint diseases (15.2% in urban vs. 6.4% in rural), chronic heart disease (17.4% in urban vs. 14.4% in rural), and stroke (13.4% in urban vs. 12% in rural) practiced yoga than did adults from rural areas suffering from these chronic conditions. Those with high cholesterol levels had a higher prevalence of yoga practice in rural areas (28%) than in urban areas (21%).

**Table 4:** Rural–urban differences in the Percentage of older adults practicing yoga/meditation/asanas/pranayama etc. more than once a week according to self -reported morbidities, India, LASI Wave 1, 2017–2018.

Background characteristics	Rural		Urban	
	% (N = 46,814)	95% CI	% (N = 25,402)	95% CI
Self-reported chronic conditions				
Hypertension or high blood pressure	13.0	12.1, 13.9	15.9	14.2, 17.8
Diabetes or high blood sugar	14.1	12.6, 15.7	14.9	12.4, 17.8
Cancer or a malignant tumour	12.7	7.7, 20.0	12.6	6.9, 21.9
Chronic lung disease such as asthma, COPD, bronchitis	9.6	8.2, 11.2	10.1	7.2, 14.1
Chronic heart diseases/congestive heart failure/other chronic heart problems	14.4	11.8, 17.4	17.4	13.0, 23.0
Stroke	12.0	9.1, 15.7	13.4	9.4, 18.7
Arthritis or rheumatism, Osteoporosis or other bone/joint diseases	9.6	8.7, 10.7	13.6	11.4, 16.0
Any neurological/psychiatric problems such as depression, Alzheimer’s/Dementia, unipolar/bipolar disorders, convulsions, Parkinson’s etc.	6.4	4.8, 8.5	15.2	10.8, 21.1
High cholesterol	28.1	23.6, 33.1	21.0	17.0, 25.7
Self-reported Pain	9.8	9.2, 10.5	16.1	14.8, 17.4
Self-rated health				
Good health	9.4	9.0, 9.8	15.1	13.6, 16.7
Poor health	8.4	7.5, 9.3	10.7	9.2, 12.5
Difficulty in daily living activities				
ADL	8.6	7.7, 9.5	12.7	10.8, 14.9
IADL	7.7	7.2, 8.3	9.7	8.5, 11.0

COPD: Chronic Obstructive Pulmonary Disease; ADL: Activities of daily living; IADL: Instrumental activities of daily living.

Approximately 10% of the adults from rural areas and 16% from urban areas reported experiencing pain from practicing yoga. Irrespective of good or poor health, a higher percentage of older adults from urban areas practiced yoga than those from rural areas did. There were 8.6% and 7.7% older adults from rural areas suffering from ADL and IADL, respectively, who practiced yoga. The corresponding prevalence in urban areas was 12.7% for ADL and 9.7% for IADL.

Table 5 presents the results of the logistic regression analysis of yoga practice performed more than once a week among middle-aged and older Indian adults. In rural areas, older adults aged 65 + years were more likely to practice yoga (AOR: 1.33, CI: 1.22–1.46) compared to adults aged 45–54 years. A similar pattern of practising yoga was observed in the urban areas (AOR: 1.29, CI: 1.16–1.43). Female older adults were 15% and 9% less likely to practice yoga in rural (AOR: 0.85; CI: 0.78–0.92) and urban areas (AOR: 0.91; CI: 0.82–1), respectively. Those with an education of ten years and above were 2.3 and 2.1 times higher likely to practice yoga in rural (AOR: 2.28; CI: 2.07–2.52) and urban (AOR: 2.13; CI: 1.91–2.37) areas compared to their uneducated peers, respectively.

**Table 5:** Logistic regression odds ratio of practicing yoga/meditation/asanas/pranayama etc. more than once a week by background characteristics among middle aged and older adults, India, LASI Wave 1, 2017–2018.

Background characteristics	Rural		Urban	
	AOR	95% CI	AOR	95% CI
Age (in years)				
45–54				
55–64	1.19***	(1.1, 1.29)	1.25***	(1.14, 1.36)
65+	1.33***	(1.22, 1.46)	1.29***	(1.16, 1.43)
Sex				
Male				
Female	0.85***	(0.78, 0.92)	0.91*	(0.82, 1)
Education				
No education				
Less than 5 years complete	1.21***	(1.09, 1.35)	1.11	(0.95, 1.29)
5–9 years complete	1.53***	(1.41, 1.66)	1.32***	(1.18, 1.47)
10 or more years complete	2.28***	(2.07, 2.52)	2.13***	(1.91, 2.37)
Marital Status				
Currently Married				
Widowed	0.63***	(0.48, 0.83)	1.46**	(1.03, 2.07)
Others	0.64**	(0.46, 0.9)	1.08	(0.73, 1.59)
Living arrangement				
Living alone				
Living with spouse	0.65***	(0.47, 0.88)	1.89***	(1.27, 2.83)
Living with spouse and children	0.79	(0.58, 1.08)	2.29***	(1.54, 3.4)
Living with children and /or others	0.99	(0.81, 1.2)	1.36**	(1.05, 1.77)
Religion				
Hindu				
Muslim	1.15**	(1.03, 1.28)	0.61***	(0.54, 0.69)
Christian	1.26***	(1.11, 1.43)	0.99	(0.85, 1.15)
Others	5.85***	(5.29, 6.47)	1.87***	(1.62, 2.16)
Caste				
SC				
ST	0.64***	(0.57, 0.72)	0.88	(0.74, 1.06)
OBC	0.91*	(0.83, 1.001)	1.01	(0.89, 1.14)
Others	1.00	(0.91, 1.1)	1.39***	(1.24, 1.57)
Current work status				
Currently Working				
Ever worked but currently not	0.97	(0.89, 1.06)	1.14***	(1.03, 1.26)
Never Worked	1.25***	(1.15, 1.37)	1.09	(0.98, 1.21)
MPCE quintile				
Poorest				
Poorer	1.18***	(1.05, 1.33)	1.35***	(1.19, 1.54)
Middle	1.44***	(1.29, 1.62)	1.59***	(1.4, 1.8)
Richer	1.69***	(1.52, 1.89)	1.72***	(1.51, 1.95)
Richest	1.69***	(1.51, 1.89)	2.03***	(1.79, 2.31)
Self-reported chronic conditions				
Hypertension or high blood pressure	1.24***	(1.15, 1.33)	1.09**	(1, 1.17)
Diabetes or high blood sugar	1.1*	(0.99, 1.22)	1.04	(0.95, 1.14)
Cancer or a malignant tumour	0.91	(0.6, 1.37)	0.97	(0.66, 1.42)
Chronic lung disease such as asthma, COPD, bronchitis	0.97	(0.84, 1.11)	0.87	(0.74, 1.04)
Chronic heart diseases/congestive heart failure/other chronic heart problems	1.18*	(0.99, 1.4)	1.15*	(0.98, 1.35)
Stroke	0.89	(0.69, 1.15)	1.06	(0.82, 1.37)
Arthritis or rheumatism, Osteoporosis or other bone/joint diseases	0.91*	(0.83, 1)	0.96	(0.86, 1.06)
Any neurological/psychiatric problems such as depression, Alzheimer’s/Dementia, unipolar/bipolar disorders, convulsions, Parkinson’s etc.	0.69***	(0.53, 0.88)	0.98	(0.77, 1.24)
High cholesterol	1.31***	(1.1, 1.55)	1.06	(0.92, 1.23)
Self-reported Pain	1.39***	(1.3, 1.49)	1.53***	(1.42, 1.66)
Self-rated health				
Good health				
Poor health	0.79***	(0.72, 0.87)	0.64***	(0.57, 0.71)
Difficulty in daily living activities				
ADL	0.96	(0.86, 1.06)	1.01	(0.90, 1.14)
IADL	0.87***	(0.81, 0.94)	0.84***	(0.76, 0.92)
Constant	0.06***	(0.05, 0.09)	0.03***	(0.02, 0.04)

AOR: Odds ratio adjusted for all the selected covariates; CI: Confidence interval; *p < 0.05; **p < 0.01; ***p < 0.001; SC: Scheduled caste; ST: Scheduled tribe; MPCE: Monthly Per capita Consumption Expenditure; COPD: Chronic Obstructive Pulmonary Disease; ADL: Activities of daily living; IADL: Instrumental activities of daily living.

Table 6 presents a detailed decomposition of the rural–urban gap in the prevalence of practicing some form of yoga among older adults more than once a week according to the exposure variables. The positive contribution of a covariate indicates that the particular covariate contributed to the widening of the rural–urban gap in the prevalence of practicing some form of yoga among respondents more than once a week, whereas the negative contribution of the covariate indicates a diminishing gap. Approximately 49.3% of the differences in the rural–urban prevalence of some form of yoga among the participants were explained by the differences in the distribution of exposure variables. Participants’ age, sex, and marital status played a negligible role in reducing the rural–urban gap in the prevalence of any form of yoga practice among older adults for more than once a week. The largest contribution to diminishing the gap in the practice of yoga among participants was education (44.2%), caste (2.5%), and chronic diseases such as hypertension (4.53%), diabetes (1.71%), high cholesterol (3.08%), and self-reported pain (5.76%). Religion and the MPCE quintile positively contributed to widening the rural–urban gap in the prevalence of any form of yoga practice among older adults more than once a week.

**Table 6:** Decomposition of rural–urban gap in practicing yoga/meditation/asanas/pranayama etc. more than once a week by background characteristics among middle aged and older adults, India, LASI Wave 1, 2017–2018.

Prob (rural)	0.103172		
Prob (urban)	0.155227		
Difference	−0.05205		
Total explained	−0.02568		
Total explained (% contribution)	49.3		

Background characteristics	Coef.	p > z	% contribution
Age	−0.00017	0.022	0.34
Sex	−0.00015	0.055	0.29
Education	−0.02302	0.000	44.22
Marital status	−0.00054	0.000	1.03
Living arrangement	−0.00002	0.714	0.03
Religion	0.00222	0.000	−4.26
Caste	−0.00131	0.008	2.51
Working status	−0.00242	0.000	4.64
MPCE quintile	0.00175	0.000	−3.35
Hypertension or high blood pressure	−0.00236	0.000	4.53
Diabetes or high blood sugar	−0.00089	0.217	1.71
Cancer or a malignant tumour	0.00006	0.426	−0.11
Chronic lung disease such as asthma, COPD, bronchitis	−0.00001	0.704	0.01
Chronic heart diseases/congestive heart failure/other chronic heart problems	−0.00034	0.175	0.65
Stroke	0.00011	0.345	−0.20
Arthritis or rheumatism, Osteoporosis or other bone/joint diseases	0.00010	0.155	−0.20
Any neurological/psychiatric problems such as depression , Alzheimer’s/Dementia, unipolar/bipolar disorders, convulsions, Parkinson’s etc.	0.00027	0.003	−0.51
High cholesterol	−0.00160	0.000	3.08
Self-reported Pain	0.00300	0.000	−5.76
Poor self-rated health	0.00020	0.007	−0.39
ADL	0.00007	0.237	−0.13
IADL	−0.00063	0.024	1.22

MPCE: Monthly Per capita Consumption Expenditure; COPD: Chronic Obstructive Pulmonary Disease; ADL: Activities of daily living; IADL: Instrumental activities of daily living.

## Discussion

Yoga, with its origin in India, has found importance worldwide because of its far-reaching effects on human health and the mind, and has now been globally recognized. However, few studies have highlighted the prevalence of yoga and meditation, particularly among middle-aged and older adults in rural and urban settings. Based on country-representative data, this study found that 14.4% of middle-aged and older adults in urban areas and 9.2% in rural areas engaged in yoga and mindfulness activities more than once per week. A study found that 11.8% of the Indian population practiced yoga in both rural and urban areas^33^. There was a higher prevalence of practicing yoga and meditation among older adults aged 55–64 years in urban areas. Middle-aged and older adults who had never worked in rural areas, and those who had worked but were not currently working in urban areas, practiced yoga and mindfulness. A higher percentage of people from urban areas with chronic conditions such as hypertension and high cholesterol practiced yoga and mindfulness activities than participants from rural areas with similar conditions.

The findings of our study suggest that adults aged 65 years or older are more likely to practice yoga and mindfulness activities than are adults aged 45–54 years. In the United States, people in younger age groups, females, those with higher educational status, higher earning and good health status, and non-Hispanic whites reported a higher prevalence of yoga practice^34^. In the United Kingdom, higher age, female gender, degree of education, non–manual labor social class, better self-rated general health, inactive occupation, and higher moderate-to-vigorous physical activity predicted yoga practice^35^. In another study specifically focusing on the urban population in eastern India, the prevalence of yoga practice was higher among females, and those who were highly educated^36^. As documented, in Indian society, males are the primary income generators, and females stay at home to look after the family and have more time to spend on personal matters, including leisure time and physical activities^37^, which may explain the increased prevalence of yoga and mindfulness practices among females in this study.

A higher probability of practicing yoga and mindfulness activities was observed in older adults with an educational level of more than ten years in both rural and urban areas than in those with no formal education. Increased education among middle-aged and older adults means that they are more informed about all spheres of life, including the preventive and curative aspects of yoga for health. Education can also be directly linked to a healthy lifestyle, awareness, and increased health-seeking, which may explain the higher prevalence of yoga and mindfulness practices among educated people^38^. Similarly, religion and household economic quintiles also positively contributed to widening the rural–urban gap in the prevalence of practicing yoga and mindfulness activities. Higher economic status was positively associated with an increased prevalence of yoga and mindfulness activities among rural and urban populations. A detailed decomposition of the rural–urban gap in the prevalence of yoga practices and mindfulness activities among respondents by various exposure variables suggests that diminishing the gap in the practice of yoga and mindfulness activities in urban and rural areas is possible by increasing the level of education, reducing the differences across castes, and addressing NCDs.

Positive associations between several chronic conditions such as hypertension, diabetes, heart disease, psychiatric disorders, and high cholesterol with yoga and mindfulness practices in rural and/or urban areas may be explained by the possible reverse causality, in which older adults with those chronic conditions might have been advised to perform yoga or mindfulness activities by doctors or health professionals. Similarly, yoga has been suggested as a potent instrument for reducing the risk of NCDs in previous studies^20,39^. The negative association between psychiatric disorders and yoga practice is also consistent with earlier findings of multiple studies suggesting yoga-related activities as therapies that can reduce the risk of psychiatric illnesses^40–43^. A large contribution of NCDs, including high blood pressure, diabetes, and high cholesterol, as well as self-reported pain, to the rural–urban differences in the prevalence of yoga and mindfulness practices were observed in this study. This suggests that people with chronic medical conditions may be recommended to practice yoga and mindfulness activities by doctors or health professionals to reduce their risk of comorbidities. In contrast, poor self-rated health and functional difficulties were negatively associated with yoga and mindfulness practice in this study. This finding supports earlier evidence that people with poor perceived health may not participate in yoga-related activities^44^. The finding also supports the evidence of reverse causality in which people who practice yoga may have better health outcomes including a better self-perceived health and functional ability^45,46^.

Within the literature, it is apparent that yoga and mindfulness offer valuable perspectives for healthcare professionals in the management and treatment of various mental and physical health disorders^47^. Several clinical studies have demonstrated promising results regarding yoga in the management of type 2 diabetes mellitus^48,49^, cardiovascular complications^50^, gastrointestinal symptoms in irritable bowel syndrome^49,51^, hypertension^50^, dyslipidemia in patients with type 2 diabetes mellitus^52^, cognitive impairment and dementia^53^, menopausal symptoms^54^, and the enhancement of the physical capabilities of healthy senior adults^49^. Additionally, yoga has been found to have a positive impact on the treatment of various mood disorders including depression^55–57^, stress^58,59^, and anxiety^60,61^.

Yoga and mindfulness practices, which have numerous documented health benefits, are determined by a suite of sociodemographic, socioeconomic, and spatial factors. A recent study on the knowledge, attitudes, and practices of yoga in India based on participants’ implicit assumptions affirmed this^62^. Key findings in the study revealed that more males than females think that yoga can help change one lifestyle^62^. With respect to age, this study found a positive association between increasing age and the belief that yoga changes one’s lifestyle. Generally, the converse was true with respect to socioeconomic strata (i.e., low, upper-low, lower-middle, upper-middle, and upper)^62^. Lastly, there was a marginal disparity between urban and rural residents regarding the belief that yoga can change one's lifestyle, with a slightly higher proportion of urban residents holding this view than their rural counterparts^62^.

In India, although there is consensus that Ayurveda and Yoga can contribute to the non-pharmacological management of many lifestyle diseases, some people could harbour scepticism towards these practices^63^. Many people may not be aware of the beneficial effects of healthy and active lifestyles including yoga and mindfulness. Some might consider yoga and mindfulness practices old, unscientific, and dogma-based. Therefore, generating evidence on the Indian knowledge system and traditional practices, including yoga, and increasing public awareness are required. The differences in practicing yoga in rural and urban areas, with an increased prevalence in urban areas, which is mainly explained by the level of education, household economic status, and chronic NCDs, have important implications for policy and practice. Moreover, the relationship between socioeconomic status and yoga needs to be explored in future research.

It is also probable that urbanization in India may be connected to yoga in one way or another because of an increase in chronic diseases. Chronic diseases may prompt individuals to resort to yoga and mindfulness. In light of this, it is imperative that measures be taken to prepare for the potential increase in chronic diseases by adopting preventive techniques such as yoga and mindfulness. On a global scale, health promotion initiatives have been implemented to foster the practice of yoga and mindfulness, such as the United Nations General Assembly's decision on December 11, 2014, which resolved to designate June 21 as the International Day of Yoga.

The findings of this study have significant policy implications for promoting the health and well-being of middle-aged and older adults through the integration of yoga and mindfulness practice. Policies could be aimed at designing therapeutic landscapes (i.e., community parks and other public spaces) that enhance accessibility and create more attractive spaces for yoga and mindfulness activities for older adults. There is also a need to advocate for the integration of yoga and mindfulness from a life-course approach, beginning in early childhood and continuing into adulthood. This also requires a combination of policies and programs, such as; the incorporation of yoga and mindfulness into physical education curricula or extra-curricular activities; promoting a culture of overall wellness in the classroom by implementing yoga and mindfulness practices, providing training programs for educators to incorporate yoga and mindfulness techniques into their teaching methods; offering educational resources for parents to understand the benefits of yoga and mindfulness for their children; and introducing financial incentives in the form of tax waivers for organizations and businesses that adopt yoga and mindfulness programs.

Furthermore, based on the results of the current study, it is apparent that yoga and mindfulness are less prevalent among less-educated older adults. Consequently, the implementation of policies concerning health literacy in relation to these activities is warranted. These policies could be tailored to increase access to yoga and mindfulness information through public awareness campaigns, increase the understanding of yoga and mindfulness information through plain language materials, and use clear communication strategies between educators and healthcare providers for older adults to make informed decisions on adopting yoga and mindfulness practices.

This study has certain limitations. Importantly, the findings have limited interpretability as the study is based on a cross-sectional design that does not allow causal inferences. It is possible that those who engage in yoga and mindfulness activities are more likely to have better physical and functional health outcomes and fewer chances of suffering from NCDs. Another important limitation is the possibility of selection bias; as education is an important component of yoga and mindfulness activities, a major proportion of the older respondents had no formal education in this study (46%); thus, the findings should be interpreted and generalized with caution. Indeed, the self-reported nature of the variables, including yoga, mindfulness practices, and health outcomes, may be subject to reporting and recall biases that can influence the current findings.

Moreover, the study considered yoga practice if someone was involved in yoga once per week. The study also failed to specifically measure the amount (level) of yoga practice and could not determine whether one’s involvement in yoga practice was adequate to match the level of physical activity recommended by the World Health Organization (WHO). The daily time duration and intensity of yoga and mindfulness practice have not been specified. Hence, from the perspective of physical activity, these critical components should be considered in future studies. Another limitation of this study is that the data were gathered before the COVID-19 pandemic, and there could have been substantial changes in the lives of people after the pandemic. Therefore, future studies should be conducted in this regard.

## Conclusions

The findings suggest that middle-aged and older adults in urban areas practice yoga and mindfulness activities more than those in rural areas do. Levels of education, household characteristics, and health outcomes such as chronic conditions, pain, and physical and functional health explain the observed differences in yoga and mindfulness practices across rural and urban areas. Age-appropriate healthy practices such as yoga and mindfulness should be encouraged to enhance the physical and mental well-being of middle-aged and older adults. Further studies are required to understand the different aspects of the middle aged and older adults’ health, be it physical, emotional, mental or cognitive that is affected by the practice of yoga, meditation, asana and pranayama.

## Data Availability

The study used secondary data which is available at the Gateway to Global Aging Data (https://g2aging.org/).
